# A web-based education program to encourage organ donation registration among lower-educated adolescents in the Netherlands: study protocol for a cluster randomized controlled trial

**DOI:** 10.1186/s13063-018-2927-6

**Published:** 2018-10-01

**Authors:** Esther Steenaart, Rik Crutzen, Math J J M Candel, Nanne K de Vries

**Affiliations:** 1 Department of Health Promotion, CAPHRI Maastricht University, Maastricht, The Netherlands; 2 Department of Methodology and Statistics, CAPHRI Maastricht University, Maastricht, The Netherlands

**Keywords:** Intervention, School, Organ donation, Registration, Adolescents, Internet

## Abstract

**Background:**

The gap between the supply and demand of organ donors is substantial, causing patients to suffer from long waiting times. Moreover, the lack of registrations places a burden on family members and medical professionals when an unregistered individual dies. This article describes a cluster randomized controlled trial (CRCT) study to assess the effectiveness and quality of implementation of a web-based program aimed at encouraging lower-educated adolescents to register a well-informed choice about organ donation, regardless of it being as a donor or not.

**Methods/design:**

The program will be delivered by teachers at schools for Intermediate Vocational Education in the Netherlands. The effectiveness will be assessed in a CRCT design with post-test only using self-administered questionnaires for the primary outcome (i.e. intention to register). Classes will be matched to improve equivalence of groups. From each pair of matched classes, one class will be randomly assigned to the experimental condition, and the other assigned to the control condition. Students in the control groups will fill in the questionnaire before receiving the program, while the experimental groups will do this afterwards. A post-test design prevents the risk of testing bias. The required sample size is 14 schools, with 10 classes per school and 13 unregistered students per class. The questionnaire assesses demographics, behavioural determinants (attitude, self-efficacy, knowledge and social outcomes), intention to register (as a donor) and registration status. Six months after delivery, registration status will be assessed again. Additionally, a process evaluation will be conducted to evaluate the quality of implementation using both qualitative (i.e. semi-structured interviews) and quantitative (i.e. logbooks, questionnaires, Google Analytics to track user behaviour at the website) methods.

**Discussion:**

Findings of the study can help to further improve the program and serve as a basis for a solid dissemination plan. Moreover, the study will provide insight into (change in) determinants of registration and donorship and the translation of research into practice of school-based health promotion interventions, which can serve as an example for others.

**Trial registration:**

The Dutch Trial Register, NTR6771. Registered on 24 October 2017. This is version 2 of the protocol (5 November 2017).

**Electronic supplementary material:**

The online version of this article (10.1186/s13063-018-2927-6) contains supplementary material, which is available to authorized users.

## Background

Despite an increase in the number of organ donors and successful transplantations in recent years, there is still a substantial gap between the supply and demand for organ donors. As a result, patients suffer from long waiting times or even die while waiting for an organ [1].

One of the many efforts to increase the number of registrations is a yearly initiative to encourage 18-year-olds to register their choice (irrespective of whether they want to be a donor or not). In the year after adolescents turn 18, they receive a letter from the Ministry of Health, Welfare and Sport asking them to register their choice. This led to a response rate of 33.0% in 2016, of which 73.9% gave permission for organ donation [2]. In other words, many adolescents did not make a choice regarding organ donation yet, or at least did not register this choice. This lack of registration leads to several problems, including the burden on family members and medical professionals when an unregistered individual dies. For example, when no donor record of a patient is found, the next of kin will be asked for consent. This is a stressful situation for all people involved and results in a high refusal rate [3–5]. To increase the registration rate, adolescents could be supported in making a well-informed decision and be encouraged to register this choice.

Reubsaet and colleagues made an effort to educate adolescents about organ donation and registration by designing an educational program for high school students. The main aim of their program was to increase the number of registrations, not necessarily the number of organ donors. The social cognitive theory (SCT) of Bandura was the main underlying theory during the development [6]. SCT describes three important factors that influence (intentions to participate in) a certain behaviour. These include people’s expected consequences of performing a certain behaviour (*positive/negative outcome expectations*), people’s expected social reactions to performing that behaviour (*social outcome expectations*) and beliefs about their own ability to perform this behaviour (*self-efficacy expectations*). Several determinant studies and pilot tests led to the development of the three educational components of the program: (1) videos to elicit discussion about positive and negative outcome expectations of organ donation and registration, (2) working with an interactive computer program with tailored feedback, and (3) practicing with completing a registration form [7–10]. This program was proven to be effective in increasing the intention to register in a large field study [11], after which the program was translated from a paper-based version into a web-based version. Web-based programs have high potential, as they can be effective in changing behavioural outcomes with low costs [12]. Moreover, web-based programs enable one to reach a large group of people with information that can be tailored to the individual [13].

Another desirable adaptation regards the target group of the program. As the original program was only used among high-school students, lower-educated students were not reached. Lower-educated students have left high school by the age of 16 (as opposed to 17–18 for higher-educated students) and usually continue their education at Intermediate Vocational Education (IVE) schools [14]. IVE schools typically teach lower-educated adolescents from the age of 16 until their early 20s. These adolescents are all about to receive the letter about organ donation or have received it recently. The need for a program about organ donation might even be higher for this group than for high school students, as studies have shown that lower-educated individuals have a less positive attitude towards organ donation than higher-educated individuals [15–17]. Moreover, the registration rate of lower-educated individuals is significantly lower than that of higher-educated individuals [18].

Adjustments had to be made to make the website suitable for lower-educated students as well. Firstly, textual changes were made, as students at IVE schools score lower on reading competencies than high school students; 20% of the students that start their IVE trajectory score even lower than the minimal reading level required for civic integration [19]. Therefore, adjustments were made to improve text comprehension. Moreover, the number of questions in the interactive computer program was decreased by half. This selection was made based on a preparatory study in the target group [20]. This study was conducted among 405 IVE students, using a questionnaire based on previous literature, identifying important beliefs regarding organ donation and registration. The selection of beliefs was based on their association strength with registration behaviour and intention and the extent to which there was room for improvement, that is, the extent to which mean scores on the beliefs are in the desired direction (e.g. in favour of organ donation registration).

Adapting the web-based educational program of Reubsaet and colleagues to lower-educated students and implementing it in IVE schools throughout the Netherlands could help reach a larger group of adolescents and therefore increase the number of registrations. As the program is now web-based and applied to a different target group, it is important to study its effectiveness. This will be done in a cluster randomized controlled trial (CRCT) with post-test only in IVE schools spread over the Netherlands. In addition, a process evaluation will be peformed to determine the quality of implementation during this trial. Combining an effect and process evaluation will give insight into the effects of the program and how these effects were achieved. This article gives an overview of the different components of the program and provides a detailed protocol for the anticipated evaluation study.

## Methods/design

### Aims

The effect evaluation aims to give insight into the effect of the program on behavioural determinants, the intention to register (as an organ donor) and the actual registration behaviour itself. Attitude, self-efficacy, social outcomes and knowledge will be assessed as behavioural determinants.

With the process evaluation we aim to gain insight into how the program was implemented. The process evaluation relies on four different data sources: questionnaires from students, logbooks, interviews with teachers and user data from Google Analytics. The use of data triangulation enables different data sources to complement one another. This provides an overview of the implementation of the educational components on six levels: *reach, dose delivered, dose received, fidelity, context * and *reasons for fidelity and dose*. These levels are inspired by the model of Steckler and Linnan [21]. Additional file 1 provides an overview of all constructs used, the definitions we use in this study and the instruments used to measure them.

### Description of the intervention

The web-based program consists of three main elements: video fragments followed by group discussions, quizzes with tailored feedback and an exercise to fill in an organ donation registration form. The website is hosted by SIDVO, which is a foundation that aims to develop, evaluate and distribute organ donation education. The elements will be provided to the students during two 50-min lessons at school. At the end of the program, students are expected to be able to make a well-informed decision about organ donation registration and will be encouraged to register this choice.

Lesson 1 consists of four video fragments followed by group discussions. Each video deals with an aspect of organ donation and registration in which both positive and negative outcome expectations are included. The videos are designed to elicit discussion among students about brain death, the possibility to help other people by donating your organs, discussing the subject with others and the importance of registering in general (from the perspective of medical professionals and family members). Teachers will be provided with a manual to facilitate discussion after each video in which students have the opportunity to share their opinions and beliefs. This activity aims to increase involvement, encourage positive beliefs and counterbalance negative beliefs.

Lesson 2 combines two program elements. First, students will individually work with the website. The website provides the participants with two quizzes. One quiz will mainly focus on students’ knowledge, while the other one will focus on their opinions. The knowledge quiz reveals a score on a scale from 1 to 10 and the correct answers to the questions after completion. In the opinion quiz each question will be followed by tailored feedback. Participants will receive information that is personally relevant and fits their needs. The main goal of the quizzes is to teach participants about organ donation and registration and give them tailored feedback on misconceptions they might have. The second part of the lesson will be spent on an exercise on completing an organ donation registration form in order to enhance self-efficacy. Students will be provided with a copy of an organ donation registration form or use a digital version, whatever option is most convenient. The aim is to give students an enactive mastery experience, which increases their self-efficacy and therefore increases the intention to fill in a real organ donation registration form.

### Study design

**Fig. 1:**
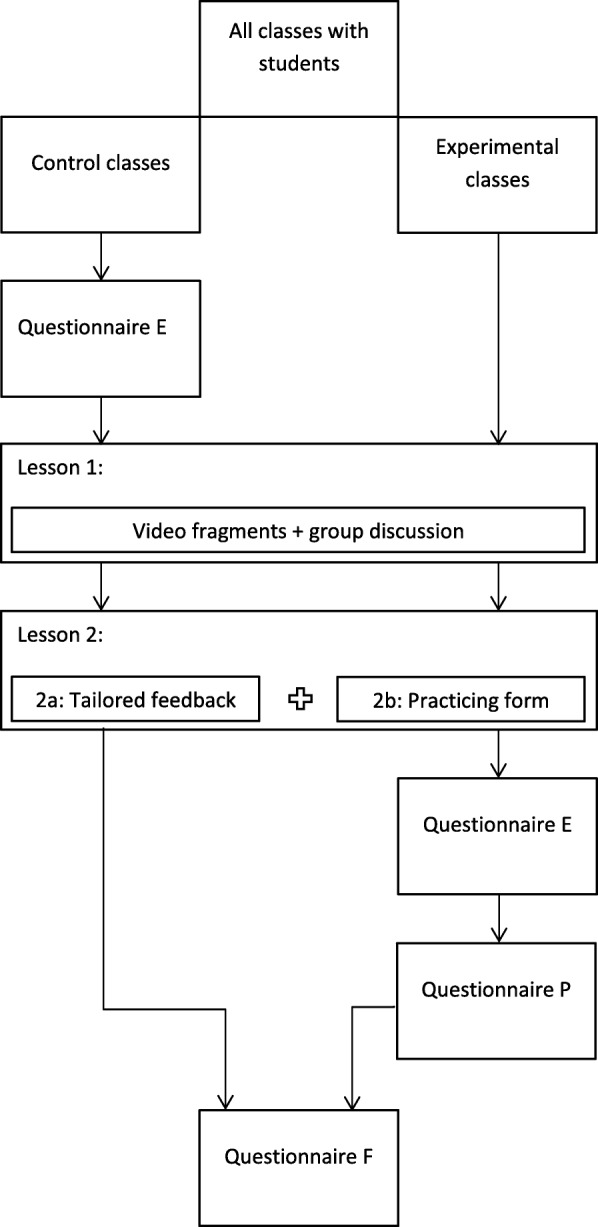
Design of the evaluation study. *Questionnaire P* questionnaire process evaluation, *Questionnaire E* questionnaire effect evaluation, *Questionnaire F* questionnaire follow-up measurement

**Fig. 2:**
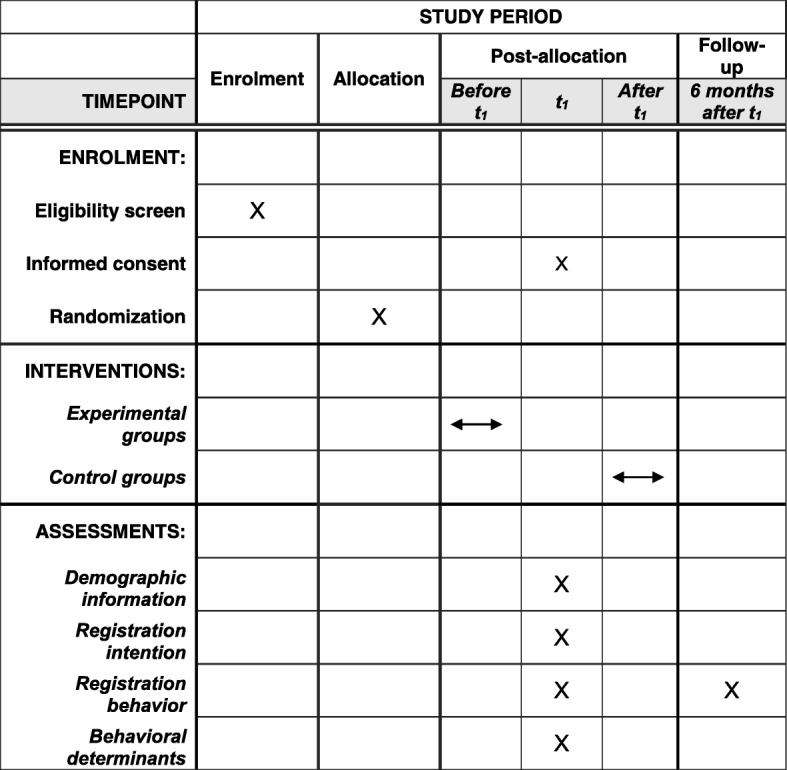
SPIRIT figure

This article was written in accordance with the Standard Protocol Items: Recommendations for Interventional Trials (SPIRIT) checklist (Additional file 2). The effects of the program on registration behaviour, registration intention and beliefs regarding organ donation and registration will be evaluated in a CRCT with post-test only (see Figs. 1 and 2). The program will be offered to both the control and experimental groups. Students in the control group will complete the evaluation questionnaire before receiving the program, while the experimental group will do this after receiving the program. Differential attrition can be prevented, as all participating classes will receive the program. A post-test design prevents the risk of testing bias by priming students for the program by filling out the questionnaire beforehand [22]. The absence of a pre-test, however, might also threaten internal validity, since equivalence of the groups cannot be established. The threat of possible differences at baseline will be minimized by using a large number of randomization units and a proper randomization [23].

After agreement to participate, classes will be matched by members of the study team to improve the equivalence of the groups. The classes will be matched on study discipline, and matched classes will be from the same school. From each pair of matched classes, one class will be randomly assigned to the experimental condition, while the other class will then be assigned to the control condition. Teachers will be notified as to whether they have to hand out the questionnaire before or after delivering the program to students.

The implementation of the educational program will be evaluated using both qualitative (i.e. semi-structured interviews) and quantitative (i.e. logbooks, questionnaires, Google Analytics data) methods, both during and post-delivery.

### Procedure

The educational program will be provided by the teachers in IVE schools during regular classes. They will receive a link via e-mail prior to the start of the study. With this link, both teachers and students can reach the website. Before the first lesson, the control group will complete the effect evaluation questionnaire. All participants will then receive lesson 1, with the video fragments and group discussions. During the next session, they will both receive lesson 2a, the quizzes with tailored feedback, and lesson 2b, the exercise on completing an organ donation registration form. Teachers will have the freedom to provide the lessons whenever it fits their schedule, but we expect that in most cases lesson 2 will be delivered within 1 or 2 weeks after lesson 1. After the whole program is delivered, students in the experimental group will fill in the evaluation questionnaire. Six months after the implementation of the program, participating students (students from both the experimental and control groups) will receive a follow-up questionnaire during class to measure their registration behaviour. New control classes will then be recruited to compare the registration behaviour of students who received the program with that of students who did not. Data for the process evaluation will be gathered partly during the program delivery and partly after. During the program delivery, data will be collected on usage (e.g. by means of Google Analytics), while after the program delivery, reasons for particular usage behaviour will be assessed (e.g. by means of interviews). Moreover, students in the experimental groups will complete a process evaluation questionnaire after the program is delivered.

### Study setting, population and sample size

The program will be evaluated in a school setting. More specifically, IVE schools throughout the Netherlands will be invited to participate in the program through various channels. In particular, teachers teaching the course ‘Citizenship’ will be approached, as organ donation fits with the content of this course. IVE schools typically teach lower-educated adolescents from the age of 16 until the early 20s, but students from all ages are welcome. Older students will therefore be automatically included in the sample as well. Students in IVE can choose from a variety of disciplines, both manual and non-manual. There are four levels within IVE, but the educational level is generally low as opposed to college or university education.

As participants will be recruited via their schools, they are not approached individually. Therefore, no inclusion or exclusion criteria are set on the individual level. On the class level, only students in IVE levels 2, 3 and 4 are included in the study. Level 1 is also known as entry training. Students in this level did not graduate from high school; therefore, we cannot guarantee they will be able to participate properly in the program due to a large variety in cognitive abilities at this level.

Data from a preparatory study in the same target group [20] and the evaluation of a similar study in a slightly different target group [11] were used in simulations to calculate the sample size for the current study. The calculations were performed using a Monte Carlo simulation study with MLPowSim and are therefore not based on design effects, but on the expected percentage of registration in the control and experimental groups as well as the variances of the random effects from the logistic multilevel model and intraclass correlations (ICCs) [24]. The calculations were based on an expected increase in the primary outcome, expected class size, the ICC and the power of the test. The matching was not taken into account, as this will only increase the power. Therefore, the chosen power level (80%) is a safe choice [25]. A relative increase of 20% of the primary outcome was minimally expected based on the results of the study by Reubsaet et al. [11]. In the preparatory study, the average number of students per class was 16.2, of which 73.8% were not registered yet (our target group). The average cluster size is therefore 11.95, or 12 unregistered students per class. In order to be able to detect a difference of 20% with 80% power in a two-sided test with α = 0.05, taking into account an ICC of 0.02 at class level and 0.01 at school level, the required sample size is 12 schools, with an average of 10 classes per school and 12 unregistered students per class, leading to a power level of 0.839. However, as there is a high range of the number of students per class, the number of schools is increased by 15% [26], resulting in a minimum of 14 schools (and thus 140 classes, of which 70 are assigned to the control condition and 70 to the intervention condition, with a total of 1680 unregistered students).

### Ethical approval

The study was approved by the Ethics Committee of the Faculty of Health, Medicine and Life Sciences on 23 October 2017 (reference number Steenaart/231017) and registered at the Dutch Trial Register (NTR6771; http://www.trialregister.nl/trialreg/admin/rctview.asp?TC=6771). Participation of teachers and students is voluntary, and they will provide written (students) or verbal (teachers) informed consent. Participants will be informed about the study, the opportunity to withdraw their consent at any time and the anonymity of the study and will be provided with contact information in case they have any further questions.

### Measurements

Four different measurement instruments will be used to gather data; questionnaires from students, logbooks, interviews with teachers and user data from Google Analytics.

#### Questionnaires

All students will participate in the evaluation study by completing a total of two or three questionnaires depending on the condition: one for the process evaluation (questionnaire P; experimental condition only), one for the effect evaluation (questionnaire E) and one follow-up questionnaire (questionnaire F). For the process evaluation, only students in the experimental condition will receive a questionnaire (questionnaire P) in which they are asked to evaluate the program after the second session. This questionnaire will assess the *dose received* and *reasons for fidelity and dose*. *Dose received* will be measured by assessing their appreciation for the program. The appreciation of the program as a whole as well as the three components will be assessed by asking the students how much they learned from each component and how much they enjoyed working with the component. Also, *reasons for fidelity and dose* will be assessed by asking students what parts they liked and what parts could be improved.

The questionnaire for the effect evaluation (questionnaire E) will be completed before (control condition) or after (experimental condition) the program delivery. The primary outcome will be the students’ intention to register (yes/no). Students who answered ‘yes’ to this question will be asked to specify the decision they would like to make (registering as a posthumous organ and tissue donor, a posthumous donor for specific organs and tissues or a non-donor; leaving the decision to the next of kin; or leaving the decision to a specific person), which will be dichotomized into intention to become a donor or not. All students will also be asked about whether they already registered a decision (yes/no) and, if yes, the choice they made (see the above-mentioned options). Only unregistered participants will fill in the intention question. Additionally, questions are included to measure attitude, self-efficacy, knowledge and social outcomes. These behavioural determinants are the main focus of the educational program.

Finally, all participants will receive a follow-up questionnaire (questionnaire F) after a period of approximately 6 months to measure their registration behaviour. As official registration records are private, participants will be asked whether they registered their choice since receiving the program and, if so, what choice they registered.

#### Logbooks

Teachers will be asked to keep track of a logbook during the two sessions. This logbook will consist of a simple checklist in which they can tick the boxes of the components they delivered during the session. Moreover, they will be asked to indicate the time spent on the components and rate their satisfaction with the lesson. Finally, there will be room for additional comments in case they encountered difficulties during the implementation of the lessons.

#### Interviews

All participating teachers will be invited to elaborate on the facilitators and barriers of implementation of the lessons during a semi-structured interview. Data from the logbooks will be used as an input for the interview guide, as well as the diffusion of innovations theory [27]. The topics that will be addressed include teachers’ experiences (e.g. ’How did you experience working with the program?’ or ’How did you like working with the manual?’), dissemination (e.g. ’Would you be interested in using the program again in the future?’) and innovation characteristics (e.g. ’To what extent does the program fit in your schools’ curriculum?’ or ’How complex was it for you to work with the program’?). The interviews are expected to last between 15 and 30 min, will be audio-recorded and will be transcribed verbatim.

#### Google Analytics

The usage of the website will be monitored by Google Analytics. This quantitative measure gives us the opportunity to assess participants’ behaviour on the website objectively. The potential of using this method in the process evaluation of Internet-delivered interventions has been demonstrated before [28]. The data will give us information on when the website is visited and by whom, how participants then behave on the website, whether they complete all elements and, if not, when they quit. This also gives us the opportunity to check whether matched classes in different arms received the program in the same period of time. As emphasized in the previous Internet-delivered interventions study [28], it is important to combine objective user data with qualitative measures. Google Analytics cannot provide an explanation for certain user behaviour, which will therefore be assessed using interviews, logbooks and questionnaires. As Google Analytics can only trace users back to their zip code, teachers will be provided with a link to be able to trace the user behaviour back to specific schools and even classes. User behaviour cannot be traced back on the individual level; only data at the aggregate class level are accessible and used for evaluation purposes.

### Statistical analyses

The first aim of this study is to investigate the effectiveness of the educational program on the intention to register a choice (i.e. the primary outcome in the study at hand, measured as yes or no). All data for the effectiveness are gathered from questionnaire E. Secondary outcomes will be the choice students intend to make (yes or no donation), behavioural determinants and finally their actual registration behaviour 6 months after receiving the intervention. The differences in outcomes between the intervention and the control group will be analysed using multilevel regression models, taking into account that students are nested within classes which in turn are nested within schools. Besides intervention group, several demographic characteristics are included in these models (i.e. sex, study discipline, age, religion and educational level), as they are expected to be associated with the outcomes.

As a post-test only design was used, there are no missing data in terms of participants dropping out in between measurements. Therefore, when comparing the experimental and control groups, missing data will not be an issue—we have all data from participants who agreed to participate. The mixed model analysis is then valid under the assumption of missing at random (MAR). However, when correcting for covariates, missing could become an issue, as participants can skip items in the questionnaire. This will be dealt with using multiple imputation (MI).

The second aim is to analyse the implementation of the program. Interviews will be audio-recorded, and a transcript will be written from each interview. The transcripts will be marked with labels using open coding. These codes will be categorized (selective coding), resulting in matrices in which facilitators and barriers of the implementation will be identified. Quantitative data from Google Analytics, the teachers’ logbooks and students’ questionnaires (questionnaire P) will be used for descriptive analyses to provide insight into how the program was used, delivered and received.

All data will be stored on a secured service and will only be accessible by the study team. A data entry check will be performed on the data from the questionnaires; range checks will also be performed. The interviews will be coded independently by two researchers.

## Discussion

This protocol describes a study to evaluate both the effectiveness and implementation of a program aimed at encouraging lower-educated adolescents to make a well-informed decision about organ donation and to register this choice. The study is strong in combining several methods and a variety of data sources. The data triangulation serves two main goals: confirmation of findings and completeness [29]. The combination of qualitative and quantitative methods is valuable, as both methods separately will not be sufficient [21]. By combining the methods, flaws can be neutralized and benefits can be strengthened [30]. This leads to a higher validity of conclusions.

There are also possible pitfalls of the study. However, by acknowledging them beforehand and taking them into account during the execution, risks can be limited. A threat during the execution of the program is the possibility of contamination. If students in the experimental group receive the program before students in the control group, students in the control group could hear about the content, biasing their answers on the evaluation questionnaire. Ideally, students from both groups (within one school) will receive the program simultaneously. However, in many cases, we expect this to be difficult due to organizational reasons and because often these classes are being taught by the same teacher. If students in the control group hear about the content before filling in the baseline questionnaire, this could influence their answers in the direction of expected intervention effects and therefore most probably lead to an underestimation of the program effect in the end.

Finally, the effectiveness of the program in terms of registration behaviour is difficult to measure. As official organ donation registration records are private, no objective data can be gathered regarding the actual registration numbers of participants. The intention to register will be measured directly after receiving the program. To gain some insight into the registration behaviour, participants will be asked about their registration status 6 months after receiving the program. The registration behaviour will thus be based on self-reported registration status. This is, however, the best possible option concerning the circumstances.

The findings of the study can help further improve the program and function as a guideline for a solid dissemination plan. Results from the process evaluation will show how the program was implemented and how this can be improved in the future. The interviews with teachers give further details on circumstances that should be taken into account when disseminating the program to a larger scale. Moreover, the study will provide insight into the translation of research into practice of school-based health promotion interventions, which can serve as an example for others.

### Trial status

Currently, the trial is ongoing. Recruitment of classes started at the end of 2017 and was completed in June 2018.

## Additional files

Additional file 1: Evaluation framework. (DOCX 21 kb)

Additional file 2: SPIRIT checklist. (DOC 122 kb)

## Abbreviations

CRCT: Cluster randomized controlled trial
IVE: Intermediate Vocational Education
