# Supplementary material for: A web-based education program to encourage organ donation registration among lower-educated adolescents in the Netherlands: study protocol for a cluster randomized controlled trial
Source: Trials. 2018 Oct 1;19:532. doi: 10.1186/s13063-018-2927-6 (PMC6167828; doi:10.1186/s13063-018-2927-6)
Supplement: Supplementary file 1 — Evaluation framework. (DOCX 21 kb) [file 13063_2018_2927_MOESM1_ESM.docx]

*Evaluation framework*

| **Effect evaluation** | | |
| --- | --- | --- |
| **Construct** | **Definition** | **Measurement instrument** |
| *Intention* | - Intention to fill in an organ donation registration form - Intention to become an organ donor | Questionnaire E |
| *Attitude* | (Mis)beliefs regarding organ donation and –registration | Questionnaire E |
| *Self-efficacy* | - The extent to which students feel confident in having enough knowledge to make a decision - The extent to which students find it difficult to discuss the topic with others | Questionnaire E |
| *Knowledge* | The extent to which students perceive their knowledge about organ donation and whether they are interested in learning more | Questionnaire E |
| *Social outcomes* | The extent to which students intend to discuss organ donation and –registration with others:   - Intention to discuss with friends - Intention to discuss with parents | Questionnaire E |
| *Registration behavior* | Actual registration of a participant | Questionnaire F |

| **Process evaluation** | | |
| --- | --- | --- |
| **Construct** | **Definition** | **Measurement instrument** |
| *Reach* | The extent to which the target group is reached:   - Number of schools reached - Number of participants reached | Google Analytics |
| *Dose delivered* | The proportion of program components actually delivered by teachers:   - Number of components delivered | Logbooks teachers  Interviews teachers |
| *Dose received* | The extent to which students engage with the program:   - Active participation during the lessons - Appreciation of the program (components) - Completing quizzes - Watching full videos | Logbooks teachers  Interviews teachers  Questionnaire P  Google Analytics |
| *Fidelity* | The extent to which the program was implemented as intended:   - Compliance to the manual - Completeness of deliverance of the components | Logbooks teachers  Interviews teachers  Google Analytics |
| *Context* | Environmental aspects that could affect the implementation:   - Compatibility with the existing curriculum - Teachers’ capacity to implement the program - Support by the schools’ board or colleagues | Interviews teachers |
| *Reasons for fidelity and dose* | Reasons for (not) implementing the program (components) as intended:   - Facilitators to implementation - Barriers to implementation | Logbooks teachers  Interviews teachers  Questionnaire P |

*Questionnaire P: Questionnaire process evaluation
Questionnaire E: Questionnaire effect evaluation
Questionnaire F: Questionnaire follow-up measurement*
